# Palmar Cooling as a Thermoregulatory Intervention: Mechanistic Basis and Implications for Exercise and Clinical Applications

**DOI:** 10.1155/tsm2/4204958

**Published:** 2026-10-18

**Authors:** Robert J. Heins, William E. Reisinger, Brett Norris, Robert Rogers

**Affiliations:** ^1^ College of Osteopathic Medicine Kansas City University 1750 Independence Avenue Kansas Missouri 64106 USA kcumb.edu; ^2^ Heritage College of Osteopathic Medicine Ohio University 191 W Union St Athens Ohio 45701 USA ohio.edu

**Keywords:** exercise performance, heat induced fatigue, heat stress, palmar cooling, thermoregulation

## Abstract

During exercise, rising metabolic heat production creates competition for blood flow between skeletal muscle and the cutaneous vasculature. The resulting redistribution elevates cardiovascular (CV) strain and accelerates fatigue, particularly during environmental heat stress or in individuals with impaired thermoregulatory capacity. Strategies that enhance heat dissipation without further burdening CV function may therefore improve exercise tolerance. Palmar cooling (PC) is an intervention that targets the glabrous skin of the hands, where dense arteriovenous anastomoses facilitate high‐flow, low‐resistance blood flow and efficient convective heat exchange, thereby augmenting systemic heat dissipation through the peripheral circulation. Evidence in healthy and athletic populations suggests that PC can decrease thermal strain while improving endurance capacity, resistance training volume, and perceptual responses when applied under meaningful heat stress, although current findings remain heterogeneous. Given age‐ and disease‐related reductions in thermoregulatory and CV reserve, PC may have translational relevance for enhancing exercise tolerance during rehabilitation, reducing heat‐related illness risk, and supporting individuals with cardiopulmonary disease. Improved within‐session tolerability may, in turn, facilitate greater cumulative training stimulus and adherence, with potential downstream benefits for overall exercise capacity. This narrative review discusses the thermoregulatory, CV, and exercise physiology underlying PC, evaluates the current evidence, and outlines future directions to support clinical translation.

## 1. Introduction

An individual’s ability to perform at their highest level is partially modulated by their ability to manage metabolic heat [1]. During physical activity, rising body temperature requires the cardiovascular (CV) system to simultaneously satisfy the metabolic demands of contracting skeletal muscle and augment cutaneous skin blood flow (SkBF) to support thermoregulation [2]. A potential method to decrease thermal strain is palmar cooling (PC), a noninvasive thermoregulatory intervention designed to facilitate heat exchange in the body’s periphery. PC takes advantage of the unique physiological properties of glabrous skin, located in the palms of the hands. The skin in these areas house specialized vascular structures known as arteriovenous anastomoses (AVAs). These are low resistance/high‐flow vascular structures that bypass capillary beds, directly connecting arterioles to venules [3]. The application of a continuously cooled stimulus to the palms via a PC device (PCD) establishes a constant temperature gradient across the AVAs, promoting efficient conductive heat transfer from circulating blood prior to its return to the central circulation [4]. Therefore, PC may theoretically facilitate individuals to sustain higher workloads and delay the onset of heat‐induced fatigue. By extracting heat directly from the circulation, PC may attenuate the rise in core temperature that otherwise drives competing demands for blood flow between contracting skeletal muscle and the cutaneous circulation, thereby reducing CV strain during prolonged exertion [2, 5–8]. Rising core and brain temperature also contribute to central fatigue and impaired motor drive. Slowing this rise may in turn preserve neural drive and allow sustained effort for longer before fatigue onset [9–11].

Current literature evaluating the efficacy of PC has primarily focused on athletic performance and exercise capacity in healthy populations. Many studies have demonstrated significant improvements in high‐intensity interval training (HIIT), resistance training volume, and endurance performance when PC is applied during rest intervals or active exercise [4, 12–34]. However, the existing body of literature remains notably heterogeneous with studies showing discrepancies in environmental conditions, the specific cooling protocols used, and the fitness levels of the participants [27–29]. Despite methodological variability, accumulating evidence that PC favorably influences thermoregulatory responses, select CV and metabolic markers, and exercise performance in healthy adults suggests potential relevance beyond athletics in clinical and rehabilitative settings, although these clinical data are lacking.

Therefore, this review aims to discuss the mechanistic basis of PC, critically evaluate the existing evidence supporting its use, and explore PC’s hypothetical potential as a translational intervention to enhance exercise tolerance and improve clinical outcomes in rehabilitative settings.

## 2. Literature Search Strategy

This manuscript is a narrative review and was not registered prospectively. A structured literature search was conducted to identify studies evaluating PC as an exercise or thermoregulatory intervention, forming the evidence base summarized in Tables 1 and 2. Literature was identified through searches of PubMed/MEDLINE, Embase, and Scopus conducted through August 3, 2026. Search terms included combinations of the following: “palm cooling,” “palmar cooling,” “hand cooling,” “glabrous skin cooling,” “cooling glove,” “heat extraction,” “heat removal,” “pre‐cooling,” and “arteriovenous anastomoses,” combined with terms related to exercise, thermoregulation, and heat stress where applicable. Searches were limited to studies involving human subjects and restricted to the English language. Full search strings for each database are provided in Appendix A in supporting information 1. Records were screened by title and abstract, followed by full‐text assessment of remaining reports against the eligibility criteria below; this process is illustrated in Figure 1. This search identified 496 records (PubMed/MEDLINE, *n* = 209; Embase, *n* = 216; Scopus, *n* = 71); after removal of 81 duplicate records, 375 records were screened by title and abstract, of which 346 were excluded. The remaining 29 reports were sought for retrieval and all were successfully retrieved and assessed for eligibility; 8 were excluded (wrong cooling method, *n* = 2; wrong methodology, *n* = 5; and wrong article type, *n* = 1). Additional three records were identified through citation searching, all of which were retrieved, assessed for eligibility, and included without exclusion. In total, 24 studies (24 reports) were included in the review. Studies were included if they evaluated palmar or hand cooling as an intervention in the context of exercise performance, thermoregulatory physiology, or clinical populations, and reported quantitative outcomes or provided mechanistic data directly relevant to the review’s aims. Studies were excluded if cooling was applied exclusively to nonpalmar regions or if the primary focus was unrelated to thermal physiology or exercise. This structured search process applies specifically to the intervention‐evidence literature described above; background and mechanistic content elsewhere in this review (e.g., glabrous skin physiology, CV and autonomic mechanisms, and clinical population physiology) draws on the broader physiological and clinical literature and was not intended to be captured by this structured search. Risk of bias for individual studies was assessed using the Cochrane Risk of Bias 2 (RoB 2) tool for randomized studies, with the RoB 2 crossover extension applied to randomized crossover trials and the standard RoB 2 tool applied to randomized parallel‐group trials. Nonrandomized studies were assessed using the RoB in nonrandomized studies of interventions (ROBINS‐I) tool. Results of these assessments are presented in Tables S1 to S3 in supporting information 1. In addition, a GRADE certainty of evidence assessment was conducted at the outcome‐domain level, and study limitations and methodological heterogeneity are discussed throughout the text where relevant to interpretation of the evidence. The GRADE assessment was performed for the five outcome domains most commonly reported in the current PC literature: endurance performance, resistance training volume, core temperature attenuation, perceptual outcomes, and CV and metabolic markers. Certainty ratings for each domain are presented in Table 2.

**TABLE 1:** Summary of current data on palmar cooling as an intervention during exercise.

Study	Study design	Study population	Exercise and cooling methods	Outcome measures	Main findings
Grahn et al. [4]	Randomized crossover trial	Healthy males and females (*n* = 26)	Exercise: uphill walking on a treadmill; Cooling: continuous palmar cooling (PC) with negative pressure cooling device; Control: no PC	Esophageal temperature, exercise duration	Attenuated temperature rise; increased exercise duration
Hsu et al. [12]	Randomized crossover trial	Triathlon trained male athletes (*n* = 16)	Exercise: cycling; Cooling: continuous PC with negative pressure cooling device; Control: no PC	Tympanic temperature, VO_2_ and lactate, trial completion time	Attenuated temperature rise, reduced VO_2_ and lactate, shortened trial completion time
Goosey‐Tolfrey et al. [13]	Randomized crossover trial	Wheelchair athletes (*n* = 8) and healthy male athletes (*n* = 7)	Exercise: 60‐min submaximal cycling/wheelchair ergometry in heat, then timed performance trial; Cooling: bilateral hand immersion in 10°C water during recovery; Control: no cooling	Auditory canal temperature (Tac), skin temperature, heart rate, rating of perceived exertion, thermal sensation, time‐trial performance	Reduced Tac in both groups (greater in able‐bodied); improved 3‐km performance in able‐bodied; trend toward improved 1‐km performance in wheelchair athletes (ns); lower HR after cooling
Grahn et al. [14] (multiple protocols)	Randomized repeated‐measures trial (within‐subject, randomized treatment order across multiple protocols)	Healthy males (*n* = 17; subsets of *n* = 12 and *n* = 8 for additional protocols)	Exercise: treadmill walking (5.6 km/h, 9%–16% grade) in insulated military overgarments (MOPP gear) in a 41.5°C environment until esophageal temperature reached ≥ 39°C; Cooling: 60‐min post‐exercise recovery treatments comparing cooling of one hand, two hands, face, feet, or all five glabrous regions (10°C–11°C circulating water), with or without −40 mmHg subatmospheric pressure; Control: no cooling	Esophageal temperature (Tes) recovery rate, heart rate, water loss	Cooling accelerated Tes recovery vs no cooling (one hand with vacuum: 1.0 ± 0.2°C/h vs 0.4 ± 0.2°C/h control); vacuum’s added benefit was not uniform across subjects; cooling effect was additive across regions, with combined face + feet + hands cooling producing the greatest Tes decrease (1.6 ± 0.2°C in 60 min); cooling also reduced water loss and post‐recovery heart rate proportionally to treatment extent
Walker et al. [15]	Randomized crossover trial	Healthy men and women (*n* = 10)	Exercise: eight 30‐s high‐intensity intervals with 90‐s active recovery; Cooling: palm cooling with commercial device (RTX) at 15°C or refrigerant gel pack during recovery; Control: no cooling during recovery	core temperature, heart rate, VO_2_, distance, power, and subjective heat stress ratings	No significant differences between cooling and control conditions in core temperature, heart rate, VO_2_, distance, or power; device ineffective at improving performance or mitigating thermal stress
Zhang et al. [16]	Randomized crossover trial	Healthy participants (*n* = 8)	Exercise: 40‐min treadmill walking/arm curls in firefighter gear with SCBA in heat; Cooling: hand‐cooling device during 40‐min post‐exercise recovery; Control: passive seated recovery	Rectal temperature (TREC)	Lower TREC with hand cooling during last 10 min of recovery; greater reduction in core temperature from start of recovery (cooling capacity ∼144% of passive recovery)
Kwon et al. [17]	Randomized crossover trial	Resistance trained men (*n* = 16)	Exercise: four sets of bench press at 85% one rep maximum; Cooling: intermittent PC applied for 3 minutes between exercise sets Control: no PC	Number of repetitions, esophageal temperature, surface EMG root mean square, rating of perceived exertion	Increased total repetitions, lower esophageal temperature, higher total surface EMG root mean square, less perceived fatigue
Grahn et al. [18]	Controlled parallel‐group training intervention	Physically active men (*n* = 8)	Exercise: biweekly bench press (four sets fixed weight) and pull‐ups; Cooling: intermittent PC under negative pressure for 3 min between exercise sets Control: no PC	Strength (1RM), work volume	Increased bench press volume, increased pullup volume, increased bench press strength (1RM)
Scheadler et al. [19]	Randomized crossover trial	Healthy men and women (*n* = 12)	Exercise: time‐to‐exhaustion running at 75% VO_2_ max in heat (30°C, 50% relative humidity); Cooling: continuous palm cooling with BEX Runner device during run; Control: no PC	Time to exhaustion, core temperature rate‐of‐rise, heart rate, RPE, Feeling Scale	No difference in core temperature rate‐of‐rise between conditions; time to exhaustion was shorter with cooling than control; no difference in total exercise time when warm‐up included
Caruso et al. [20]	Randomized crossover trial	Healthy men and women (*n* = 35)	Exercise: four‐set leg press workout, performed under 3 conditions; Cooling: intermittent palm cooling (15°C) between sets, or between sets plus post‐exercise; Control: no PC	Skin temperature, blood lactate, average power	Palm cooling hastened heat removal and blood lactate clearance; delayed decline in average power (greatest benefit with between‐set cooling); effects varied by cooling protocol and were more pronounced in “high responder” subgroup
Kwon et al. [21]	Randomized crossover trial	Resistance trained women (*n* = 8)	Exercise: four sets of 85% one rep maximum to failure; Cooling: intermittent PC using a fixed temperature cooling plate for 3 min between exercise sets; Control: no PC	Work volume	Increased repetitions and total volume load
Adams et al. [22]	Randomized crossover trial	Physically active men (*n* = 9)	Exercise: 60 min of treadmill exercise (submaximal) in 40°C heat; Cooling: hydrated with hand cooling on both hands or dehydrated with hand cooling on both hands; Controls: hydrated with passive rest or dehydrated with passive rest	TREC, HR, cooling rate during post‐exercise recovery	Irrespective of hydration status greater reduction in TREC with bilateral hand cooling; poor effects on cooling rate and HR
Zochowski and Docherty [23]	Randomized crossover trial	Elite male swimmers (*n* = 10)	Exercise: 8 × 100 m freestyle swimming set at fastest sustainable pace; Cooling: intermittent palm cooling with RTX device during 90‐s rest intervals; Control: no cooling during rest intervals	100 m time, core temperature, heart rate, RPE, thermal comfort, thermal sensation	Faster final‐repetition 100 m time with cooling; no significant differences in core temperature, heart rate, RPE, thermal comfort, or thermal sensation between conditions
Adams et al. [24]	Randomized crossover trial	Healthy heat acclimated and nonheat acclimated males (*n* = 17)	Exercise: two 60‐min treadmill bouts (submaximal) in 40°C heat after; Cooling: 10 min of bilateral hand cooling post‐exercise; Controls: No cooling post exercise. Compared before and after heat acclimation.	TREC cooling rate	Greater cooling rate with hand cooling vs no cooling when nonheat acclimated; no difference between cooling and control once heat acclimated
Maroni et al. [25]	Randomized crossover trial	Trained male cyclists (*n* = 10)	Exercise: 30 min precooling period of cycling followed by 43‐min high intensity cycling race simulation; Cooling: Hand cooling glove, cooling jacket, or combined glove and jacket; Controls: no cooling	Core temperature, mean skin temperature, heart rate, sweat loss, total work, mean power output, cognitive performance	Combined cooling reduced skin temperature and thermal sensation during precooling; no reduction in core temperature; no improvement in total work or mean power output; no effect on cognitive performance; slightly lower sweat loss with cooling
O’Brien et al. [26]	Randomized crossover trial	Healthy males and females (*n* = 34)	Exercise: eight stages of rowing for 2 min each separated by 45–60 s of rest; Cooling: intermittent PC using gloves during exercise only and during exercise plus post‐exercise; Control: no PC	Distance rowed, HR, blood lactate, rated perceived exertion	Increased distance rowed, decrease in HR and lactate, lower perceived exertion
McMahon et al. [27]	Randomized crossover trial	Resistance trained men (*n* = 11)	Exercise: four sets of bench press at 80% one rep maximum to failure; Cooling: intermittent PC for 1 min between exercise sets; Control: no PC	Total volume load, bar velocity and force, lactate, HR, rate of perceived exertion, EMG measurements	No significant differences in any outcome measure
McMahon and Kennedy [28]	Randomized crossover trial	Resistance trained men (*n* = 11)	Exercise: four sets of bench press at 80% one rep maximum to failure; Cooling: intermittent PC for 1 min between exercise sets; Control: no PC	Total volume load, bar velocity and force, lactate, heart rate, rate of perceived exertion	No significant differences in any outcome measure
Kramer [29]	Parallel group randomized trial	Healthy males and females (*n* = 34)	Exercise: Queens College Step Test, leg extension peak force, vertical jumping maximum; Cooling: intermittent PC with a cooling glove for 2.5 min between exercise sets; Control: no PC	Power output, endurance time, strength	No significant differences in strength or power; improved endurance
Park and Kim [30]	Non‐randomized fixed order trial	Healthy males and females (*n* = 30)	Exercise: high‐intensity incline treadmill walking; Cooling: continuous cooling immediately after exercise for 20 min with cooling gloves; Control: no PC	Blood lactate concentration, heart rate recovery rate, VO_2_ max measurement, degree of recovery of muscle strength, muscular endurance, muscle fatigue	Decreased blood lactate concentration, decreased body temperature, improved cardiopulmonary function, increased muscle strength and endurance, increased degree of muscle recovery
Iwata et al. [31]	Randomized crossover trial	Healthy males (*n* = 10)	Exercise: three 20‐min cycling trials on three separate days in a hot environment; Cooling: palmar precooling for 20 min followed by continuous PC during exercise; Control: no PC	Rectal and skin temperature, heart rate, local sweat rate, VO_2_/VCO_2_, exhaustion rating, thermal sensation/comfort	No significant differences in temperature readings, HR, sweating, or VO_2_/VCO_2_ readings; cooling improved subjective thermal sensation/comfort
Brown et al. [32]	Randomized controlled trial (parallel‐group)	Healthy males and females (*n* = 15)	Exercise: repeated sprints; Cooling: three 2‐min PC bouts applied between exercise blocks; Control: no PC	Sprint time, heart rate, repetitions until exertion, muscle soreness rating (48 h after exercise)	Less degradation in sprint times, lower HR upon completion and a lower soreness rating
Wydotis et al. [33]	Randomized crossover trial	Healthy participants (*n* = 45)	Exercise: three 20‐s cycling sprints separated by 2‐min active recovery; Cooling: intermittent palm cooling during recovery; Control: no PC	Palm temperature, thermal flux, peak power (absolute and relative to body mass)	Higher thermal flux with cooling; greater peak power with cooling during second and third sprints
Wang et al. [34]	Randomized placebo‐controlled crossover trial	Recreationally trained men (*n* = 14)	Exercise: four sets of biceps curl to failure at 70% one‐repetition maximum; Cooling: hand cold‐water immersion (10°C) during 60 s of 90 s interset rest; Placebo: thermoneutral immersion with placebo induction; Control: thermoneutral immersion, no cooling	Total exercise volume, EMG amplitude, RPE, palm temperature, barbell velocity, heart rate	Greater exercise volume with cooling vs. control and placebo; placebo also improved volume vs. control; cooling increased EMG amplitude and lowered RPE from set 2 onward; no differences in barbell velocity or heart rate

*Note:* EMG: electromyography, VO_2_: volume of oxygen, VCO_2_: volume of carbon dioxide; TREC: rectal temperature, Tes: Esophageal temperature.

Abbreviations: 1RM, one repetition maximum, HR, heart rate.

**TABLE 2:** GRADE certainty of evidence assessment for palmar cooling outcomes in healthy and athletic populations^∗^.

Outcome	No. of studies	No. of participants	Reasons for downgrading	Certainty of evidence	Summary of findings
Endurance Performance	10	∼193	Risk of Bias (−1): Overall RoB ranged from low risk to some concerns; several nonrandomized designs carried moderate to serious risk; participant blinding proved unfeasible. Imprecision (−1): Small cohorts paired with heterogeneous methodologies and divergent effect estimates.	Low	Most studies performed under meaningful thermal strain demonstrated improved exercise tolerance and/or attenuated thermal strain. Benefits were less consistent in heat‐acclimated participants and under lower thermal stress, suggesting efficacy depends on environmental conditions, exercise modality, and cooling protocol.

Resistance Training Volume	9	∼115	Risk of Bias (−1): Mixed RoB across studies. Inconsistency (−1): Divergent findings across cooling durations and thermal environments. Imprecision (−1): Small sample sizes and heterogeneous interventions.	Very Low	Most studies reported increased repetitions, work volume, or strength adaptations when cooling was applied between sets. Recent studies using shorter cooling durations under thermoneutral conditions generally found no benefit, suggesting that sufficient thermal load and cooling exposure are important moderators.

Core Temperature Attenuation	11	∼136	Risk of Bias (−1): Mixed study quality with some concerns among randomized studies. Imprecision (−1): Magnitude of cooling varied by device, temperature, timing, and exercise protocol.	Moderate	The majority of studies reporting temperature outcomes demonstrated meaningful attenuation of core temperature rise during exercise with PC. Effect was absent or minimal in trained, heat‐acclimated individuals, consistent with ceiling effects in those with superior thermoregulatory reserve.

Perceptual Outcomes (RPE/Thermal Comfort)	12	∼181	Risk of Bias (−1): Subjective outcomes with participant blinding generally not feasible. Imprecision (−1): Small studies and variable outcome measures.	Low	Palmar cooling generally improved thermal comfort and/or reduced perceived exertion, although improvements were not universal and were sometimes observed despite minimal physiologic changes.

CV and Metabolic Markers (HR, Lactate)	13	∼215	Risk of Bias (−1): Mixed RoB across included studies. Inconsistency (−1): Effects on heart rate, lactate, and metabolic responses were inconsistent. Imprecision (−1): Small samples and heterogeneous exercise/cooling protocols.	Low	Evidence suggests potential reductions in heart rate, blood lactate, and CV strain under conditions of sufficient thermal stress; however, findings remain heterogeneous and protocol dependent.

Abbreviations: CV, cardiovascular; GRADE, Grading of Recommendations, Assessment, Development and Evaluations; HR, heart rate; PC, palmar cooling; RPE, rating of perceived exertion.

^∗^Risk of bias judgments were informed by the formal Cochrane Risk of Bias 2 (RoB 2), including the crossover extension where applicable, and ROBINS‐I assessments presented in supporting information 1 (Tables S1 to S3). Indirectness was not considered serious because the included studies directly evaluated palmar cooling interventions in the populations and outcomes of interest. Publication bias was not formally assessed because of the limited number of studies within each outcome domain. Certainty ratings represent an outcome‐level synthesis for this narrative review and complement the individual‐study risk of bias assessments.

**FIGURE 1:**
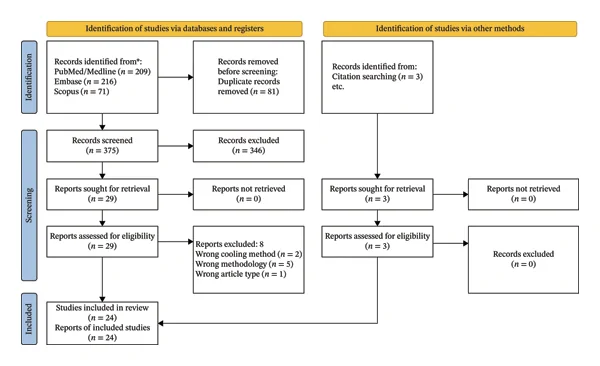
Flow diagram illustrating the identification, screening, and selection of studies included in the evaluation of palmar cooling as an exercise intervention (Table 1). Records were identified through searches of PubMed/MEDLINE, Embase, and Scopus, screened for relevance, and assessed for eligibility against the inclusion and exclusion criteria described in the Literature Search Strategy section, resulting in 24 studies included.

## 3. Glabrous Skin Physiology and Heat Exchange

While whole‐body heat dissipation is mediated primarily through generalized cutaneous vasodilation and sweating, specialized vascular structures termed AVAs play a critical role in regional heat exchange. AVAs are most abundant in the fingers and glabrous skin of the hands and feet, anatomical regions where heat transfer is particularly efficient [3, 35]. Structurally, AVAs are short vessel segments with thick muscular walls and dense sympathetic adrenergic innervation, allowing tight regulation of blood flow [36]. Like arterioles, AVAs possess a circular smooth muscle layer but also contain an additional longitudinal muscle layer that enables a sphincter‐like mechanism controlling direct arteriovenous shunting, bypassing capillary beds entirely to divert blood to superficial venous plexuses (Figure 2) [3]. Because they bypass capillary exchange, AVAs function primarily as thermoregulatory conduits, facilitating convective heat transfer from the body’s core to the skin surface rather than participating in nutrient delivery [3]. During moderate exercise, blood flow in AVA‐rich regions demonstrates a characteristic transient reduction followed by restoration toward baseline, whereas adjacent non‐AVA skin shows minimal change [37]. This pattern indicates that early exercise‐related cutaneous vasomotor adjustments occur predominantly within AVAs rather than capillary beds.

**FIGURE 2:**
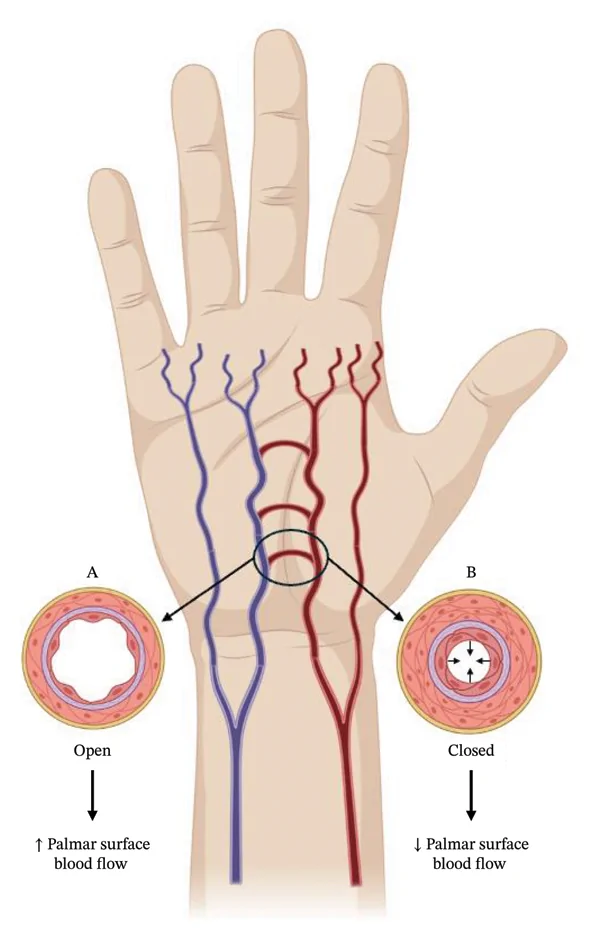
Anatomy of arteriovenous anastomoses (AVA’s) within the glabrous skin of the hand. AVAs allow for redirection of blood from arterial to venous circulation, bypassing deeper capillary beds enabling rapid modulation of surface skin blood flow for thermoregulation. When AVAs are open (A), increased palmar surface blood flow facilitates increased heat exchange. When AVAs are closed (B), less blood flow is directed to the palmar surface and further blood flow is directed to deeper capillaries decreasing heat dissipation.

AVAs exhibit marked vasomotor tone at rest under sympathetic vasoconstrictor control [3]. This maintains low basal blood flow through these shunts and directs blood instead through deeper nutritive capillary beds. This pattern limits peripheral heat loss and supports core temperature under thermoneutral conditions. During heat stress, withdrawal of sympathetic activity permits substantial dilation of these channels, resulting in pronounced increases in local blood flow and enhanced heat transfer to the periphery [3]. Due to their capacity for rapid and large changes in blood flow, AVAs function as high‐capacitance conduits that can transfer significant volumes of warm blood to the extremities, contributing disproportionately to thermoregulatory heat dissipation relative to their size. The high surface area‐to‐mass ratio of the hands and feet, combined with their dense AVA networks, further supports their role as highly efficient sites of convective and radiative heat exchange [38]. AVA‐mediated blood flow is also highly temperature dependent. Experimental observations demonstrate that during progressive local cooling of the acral skin, blood flow velocity fluctuations persist until a critical threshold of approximately 21°C–23°C, below which AVAs close abruptly and blood flow becomes uniformly low, reflecting sustained vasoconstriction [39]. Under these conditions, total cutaneous blood flow falls below basal levels as cold‐induced sympathetic activation constricts both AVAs and surrounding cutaneous vessels, preserving core temperature at the expense of peripheral perfusion. This thermal threshold behavior has important implications for external cooling strategies, as excessive local cooling may paradoxically reduce heat transfer by inducing AVA closure.

This thermal threshold behavior indicates that AVA‐mediated heat exchange is not simply proportional to the degree of cooling applied, instead it depends on maintaining local skin temperature within a defined operating range, since excessive cooling may paradoxically reduce heat transfer by inducing AVA closure. It is this capacity for large, rapid, and reversible shifts in blood flow, transitioning from near‐maximal shunting to near‐complete closure within a narrow temperature range, that makes the glabrous skin of the hands an attractive target for external cooling strategies, provided device temperatures remain above the vasoconstrictive threshold described above [3, 39].

## 4. Human Thermoregulation During Exercise

During exercise, a substantial increase in metabolic heat production results in a rise in internal body temperature [2]. Because only a fraction of the energy liberated from ATP hydrolysis is converted into external mechanical work, the majority is released as heat, creating a significant thermoregulatory burden during sustained activity [1, 40]. To prevent excessive hyperthermia, the body must dissipate the accumulated heat to maintain thermal homeostasis. Heat storage occurs when heat gain exceeds heat loss, resulting in a progressive rise in core temperature. The greatest rate of heat storage typically occurs within the first 15–20 min of moderate‐intensity steady‐state exercise, after which heat balance approaches steady state by approximately 30 min under compensable conditions as thermoeffector mechanisms become fully engaged [41].

The primary autonomic mechanisms responsible for heat dissipation are cutaneous vasodilation and sweating. Increased skin blood flow enhances dry heat exchange, primarily through convection and radiation when environmental conditions permit, whereas sweating facilitates evaporative heat loss, which becomes the dominant avenue for heat dissipation during moderate‐to‐high intensity exercise, particularly in warm environments [2, 41]. These thermoeffector responses impose stress on the CV system, as cardiac output (CO) must be distributed between active skeletal muscle and the cutaneous circulation while during prolonged exercise in the heat, reductions in plasma volume and stroke volume contribute to compensatory tachycardia [5–8]. The effectiveness of these mechanisms is influenced by environmental temperature and humidity, clothing, hydration status, age, aerobic fitness, acclimatization status, and chronic disease [2, 41, 42].

Resting core temperature in healthy adults is typically maintained near 36.5°C–37.0°C [43]. During exercise in warm or hot environments, core temperature commonly rises to the range of 38.5°C–40.0°C depending on exercise intensity, environmental conditions, and individual characteristics [2, 44, 45]. As internal temperature approaches the upper end of this range, the capacity for compensatory heat dissipation becomes increasingly strained, particularly in hot and humid conditions where evaporative heat loss is limited [46, 47]. When heat production exceeds the ability to dissipate thermal load, progressive hyperthermia impairs CV stability, central motor drive, and skeletal muscle function further contributing to earlier fatigue and voluntary exercise termination [2, 9, 44].

## 5. Integrative CV and Autonomic Mechanisms

### 5.1. Redistribution of CO, Central Hemodynamics, and Autonomic Modulation

Exercise in the heat imposes competing demands on the CV system, requiring simultaneous perfusion of active skeletal muscle and increased SkBF for heat dissipation [48]. This dual requirement substantially increases circulatory strain, as CO must be maintained through compensatory tachycardia in the setting of declining stroke volume [5–8]. Rising core temperature augments sympathetic outflow, reflected by increases in both skin sympathetic nerve activity and muscle sympathetic nerve activity, which coordinate thermoregulatory and hemodynamic responses [49, 50]. Cutaneous vasodilation facilitates heat dissipation, while sympathetic vasoconstrictor tone is maintained in nonessential vascular beds, such as the renal and splanchnic circulations, in order to preserve arterial pressure [6, 50]. Despite these compensatory mechanisms, progressive thermal strain, exacerbated by dehydration and peripheral blood pooling, reduces central blood volume and ventricular preload. The resulting decline in stroke volume necessitates a compensatory increase in heart rate to maintain CO, a phenomenon commonly described as CV drift [8]. These responses may be altered in aging and cardiopulmonary disease states, where reduced CV reserve constrains tolerance of thermal strain. Interventions that attenuate the rise in core temperature, such as PC, may therefore reduce the global vasodilatory demand on the circulation and improve hemodynamic efficiency at a given workload [4, 12].

### 5.2. Metabolic Efficiency Under Thermal Strain

Hyperthermia during exercise elevates sympathetic activation and CV strain, increasing the relative oxygen cost of a given workload. As thermal stress increases, there is a greater reliance on carbohydrate metabolism given the decline in oxygen delivery to the body’s tissues. This is driven in part by sympathetic stimulation of glycogenolysis and increased glycolytic flux [1, 51]. Concurrently, competing circulatory demands between cutaneous and muscular vascular beds may compromise optimal skeletal muscle perfusion, promoting an earlier shift toward anaerobic metabolism [52]. The result is greater lactate production for a similar external workload and earlier attainment of the lactate threshold, which can reduce exercise efficiency and shorten time to exhaustion [1, 52].

The implementation of PC may diminish these deleterious effects of thermal strain by slowing the rise in core temperature, with some studies reporting lower postexercise lactate concentrations compared to control conditions, suggesting a potential delay in anaerobic contribution and improved workload sustainability [26, 30].

### 5.3. Central Fatigue, Neural Drive, and Behavioral Regulation

Many studies in both humans and animal models suggest that core temperatures approaching 39°C–40°C represent a threshold beyond which functional capacity declines [45–47]. Rising core and brain temperatures contribute to reduced voluntary activation and central motor drive, likely serving as a protective mechanism to limit further heat storage [10, 11]. Experimental evidence demonstrates that hyperthermia is associated with reduced cortical excitability and impaired central motor output, even when peripheral contractile capacity remains preserved [9, 53]. This dissociation suggests that central fatigue during heat stress is not solely the result of peripheral muscle failure but rather reflects deliberate downregulation of motor unit recruitment to prevent critical hyperthermia. As thermal strain increases, reductions in neural drive become a major contributor to earlier exercise termination.

Elevated skin and core temperatures also significantly increase perceived exertion. Even in the presence of an otherwise compensated metabolic state, thermal discomfort disproportionately influences effort regulation [44, 54]. Afferent feedback from peripheral thermoreceptors, integrated centrally within hypothalamic and cortical networks, contributes to anticipatory adjustments in workload [11]. This has been shown experimentally where individuals often reduce intensity preemptively in response to rising thermal strain, reflecting feedforward behavioral regulation designed to avoid further heat storage [55]. By slowing the rise in core temperature, PC may preserve central motor drive, alleviate the perceptual burden of heat stress, and allow sustained effort at matched workloads for longer durations before fatigue.

## 6. Development of PC Technologies

A PCD functions as a heat sink, cooling warm arterial blood on the palmar surface of the hand and returning cool venous blood to the body’s core [4]. During submaximal exercise, the PCD dissipates heat from the body’s core, attenuating hyperthermia‐associated CV and perceptual strain and thereby supporting a higher sustained workload [4, 12]. However, excessively cold temperatures may cause vasoconstriction in the hand, limiting heat transfer [39]. Effective cooling requires a balance that minimizes vasoconstriction while allowing for maximal heat exchange [56].

The original PCD described by Grahn et al. [4] and Hsu et al. [12] was a custom‐built heat sink operating under mild negative pressure and maintained at 18°C–22°C. This temperature range was selected to promote heat extraction while minimizing cold‐induced vasoconstriction of the AVAs. The application of mild negative pressure further enhanced palmar blood flow, allowing effective heat transfer at relatively warmer surface temperatures than would otherwise be required in the absence of suction. In these studies, participants grasped a cooled bar within an isolated chamber, allowing heat to be efficiently extracted from the palmar surface during continuous walking or cycling exercises. Building on this design, subsequent studies have tested variations of this application. Some employed continuous cooling with negative‐pressure devices during exercise, while others evaluated intermittent cooling between sets of resistance exercise using similar devices, cooling plates, cooling gloves, or cooling cylinders maintained at 10°C–15°C [13–34]. More recently, companies have commercially developed PCDs in compact, low‐cost designs consisting of lightweight metal cylinders filled with mildly cold water which can be used during, between, or after training sessions. This shift away from negative‐pressure systems likely reflects practical rather than physiological considerations. The original negative‐pressure apparatus requires a sealed chamber and vacuum‐generating equipment, adding substantial cost, bulk, and setup complexity that limits its feasibility outside a controlled laboratory setting. Compact, unpressurized designs trade the theoretical blood flow augmentation provided by negative pressure for portability and affordability, but whether this trade‐off meaningfully affects cooling efficacy has not been directly tested. Most studies using nonnegative‐pressure devices (cooling plates, gloves, or cylinders) [13, 15–34] were not designed as head‐to‐head comparisons against negative‐pressure systems; it remains unclear whether negative pressure provides a necessary physiological advantage or whether comparable heat extraction can be achieved through simpler passive‐contact designs. This represents an important unresolved question for device optimization and warrants direct comparative study. One study has directly compared cooling with and without subatmospheric pressure and found that its benefit was not uniform across individuals: approximately half of the subjects showed improved heat extraction with vacuum application, while the other half showed no additional benefit [14]. This variability may partly reflect differences in baseline AVA blood flow, as athletes exercising at high intensity often already have elevated blood pressure and blood flow, which may reduce the marginal contribution of vacuum‐assisted flow augmentation. In addition, forgoing the vacuum component allows both hands to be cooled simultaneously without the added cost and complexity of a sealed chamber, and this bilateral application may offset any reduction in per‐hand flow through a doubling of the total cooling surface area.

## 7. PC Evidence in Healthy and Athletic Populations

Over the past 2 decades, PC has been investigated across endurance, resistance, and mixed‐modality exercise models, with the strongest evidence emerging under conditions of meaningful thermal strain. In endurance settings, early work by Grahn et al. [4] and Hsu et al. [12] demonstrated that continuous PC combined with mild negative pressure mitigated the rise in core temperature and extended exercise tolerance in hot environments. This was extended by Goosey‐Tolfrey et al. [13], who reported reduced auditory canal temperature and improved 3‐km time‐trial performance in able‐bodied athletes (with a similar nonsignificant trend in wheelchair athletes), and by post‐exercise recovery studies in firefighters [16] and hydrated/dehydrated exercisers [22], both showing accelerated cooling rates with hand cooling. Not all studies concur, however: Walker et al. [15] found no benefit of a commercial cooling device on core temperature, heart rate, or performance during recovery from high‐intensity intervals, and Scheadler et al. [19] found palm cooling did not slow core temperature rise during time‐to‐exhaustion running and was associated with a shorter time to exhaustion than control. Study characteristics and outcomes for all 24 included trials are summarized in Table 1.

In resistance‐trained populations, intermittent PC applied between sets has most often increased work volume, delayed fatigue, and augmented strength adaptations [17, 18, 21]. Caruso et al. [20] found that cooling hastened heat removal and lactate clearance and delayed decline in average power during leg press exercise, with effects concentrated in a “high responder” subgroup, and Wang et al. [34] recently reported greater exercise volume, higher EMG amplitude, and lower RPE with interset hand cooling producing further gains beyond a placebo effect that was itself present. Set against this, McMahon et al. [27] and McMahon and Kennedy [28] found no significant effect of PC on acute bench press performance, EMG amplitude, or metabolic markers, and Kramer [29] found no benefit to strength or power, though endurance did improve. Cooling in these null‐result studies was limited to brief (1‐ to under‐3‐min) intervals under thermoneutral conditions, which may not generate sufficient systemic heat accumulation for thermoregulation to meaningfully constrain performance; under such conditions, muscular fatigue or neural drive, rather than hyperthermia, may be the primary determinant of repetition failure. The absence of benefit in these trials therefore does not necessarily refute PC’s efficacy so much as underscore its context dependence.

More recent work has broadened the scope of application to rowing, sprinting, cycling, and mixed functional tasks, with generally favorable but methodologically uneven results. O’Brien et al. [26] reported increased distance rowed, lower heart rate and lactate, and reduced perceived exertion with intermittent cooling, the most favorable rowing outcome in this review, but this trial also carried the highest risk of bias of any crossover study assessed (RoB 2: high risk), driven by substantial attrition and inconsistent protocol completion, and should be weighted accordingly. Zochowski and Docherty [23] found a faster final‐repetition swim time despite no change in physiologic or perceptual measures; Brown et al. [32] found less sprint‐time degradation, lower heart rate, and lower 48‐h soreness with interset cooling; and Wydotis et al. [33] found greater thermal flux and higher peak power in later repeated cycling sprints. Park and Kim [30] reported improved lactate clearance, cardiopulmonary recovery, and strength/endurance recovery with post‐exercise cooling gloves; however, this study’s nonrandomized, fixed‐order design carried a serious ROBINS‐I risk of bias due to uncontrolled confounding, meaningfully limiting the causal weight of its findings relative to the randomized trials in this table.

A separate pattern emerges among trained or heat‐acclimated individuals: Iwata et al. [31] and Maroni et al. [25] found minimal or no performance benefit of hand cooling in trained cyclists despite reduced skin temperature or improved subjective comfort, consistent with Scheadler et al.’s [19] null finding in trained runners, and with Adams et al. [24], who found cooling benefited only nonheat‐acclimated participants. This suggests thermoregulatory and CV adaptations in trained or acclimated individuals attenuate the marginal benefit of peripheral heat extraction, strengthening, rather than weakening, the rationale for testing PC in populations where thermoregulation is impaired (CV disease, prior heat illness, and rehabilitation settings) rather than in elite or acclimated athletes although this has not been tested in any controlled study to date.

An additional methodological factor may also partly explain the null findings specifically in the cycling‐based protocols of Iwata et al. [31] and Maroni et al. [25]. Sustained gripping, such as maintaining hold of handlebars during cycling, applies mechanical pressure to the ulnar and median nerves and adjacent vasculature at the wrist and palm, a mechanism well documented in the development of handlebar‐associated neuropathy in long‐distance cyclists [57]. The ulnar artery courses through the same anatomical compartment (Guyon’s canal) as the ulnar nerve; thus, it is hypothesized that comparable mechanical loading could restrict local blood flow to the glabrous skin independent of thermal effects, although this vascular consequence has not yet been directly measured in cycling populations. Based on this theoretical framework, it is possible that grip‐related compression during cycling limited effective heat exchange in these studies, independent of cooling device efficacy. This remains a speculative mechanism that has not been systematically addressed in the existing PC literature, and future research is required to validate whether such mechanical interference significantly impairs AVA‐mediated cooling during cycling.

Across the 24 studies evaluated here, risk of bias assessment found most randomized trials to carry some concerns, chiefly from incomplete reporting of randomization procedures, the practical infeasibility of blinding participants to a palpable cooling stimulus, and selective outcome reporting. McMahon et al. [27] and McMahon and Kennedy [28] were the only trials rated as overall low risk; O’Brien et al. [26] was the sole high‐risk trial; and the nonrandomized Grahn et al. [18] pull‐up/strength protocols and Park and Kim [30] were rated moderate to serious risk under ROBINS‐I, largely from uncontrolled order effects. Certainty of evidence for each outcome domain, assessed using the GRADE framework, is presented in Table 2.

This is reflected in the GRADE assessment: certainty ranged from very low (resistance training volume) to moderate (core temperature attenuation), with no domain reaching high certainty. Taken together, performance benefits appear most consistent when heat accumulation meaningfully contributes to CV strain and fatigue, while brief or low‐intensity cooling under minimal thermal load is unlikely to alter performance outcomes.

## 8. Potential Clinical and Translational Applications

The applications discussed below are, with one exception, mechanistically motivated hypotheses rather than evidence‐based recommendations. No published trials have directly tested PC in older adults, patients with cardiopulmonary disease, postsurgical populations, or heat illness management; the rationale presented in these areas is extrapolated from the physiology of impaired thermoregulation in these populations and from PC’s demonstrated effects in healthy individuals, and should be interpreted accordingly. The sole exception is the application to multiple sclerosis (MS), discussed below, where a small amount of direct clinical trial evidence exists.

### 8.1. Neurological Disorders With Heat‐Sensitive Symptom Exacerbation

Among the populations discussed in this section, MS offers the clearest direct evidence for PC’s clinical relevance, rather than physiological rationale alone: demyelination of central nervous system axons alters saltatory conduction and predisposes affected fibers to conduction slowing or blocking as temperature rises, a phenomenon known as Uhthoff’s phenomenon [58]. An estimated 60%–80% of individuals with MS experience transient worsening of neurological symptoms, including visual disturbance, weakness, and fatigue, with even modest increases in core or ambient temperature [59]. These exacerbations are often reversible with cooling, interventions that attenuate the rise in body temperature during activity may meaningfully improve functional capacity and quality of life in this population.

Preliminary evidence directly supports this application serving as a powerful proof‐of‐concept for the broader clinical utility of AVAs. In a study of ambulatory, heat‐sensitive individuals with MS, unilateral PC utilizing a specialized rigid chamber with slight negative pressure (−40 mmHg) was applied during treadmill walking [59]. This reduced exercise‐related heat stress increased exercise durations by 33%–35% and delayed the onset of symptom exacerbation and subjective fatigue compared to uncooled control trials [59]. Although small and preliminary, this study provides direct proof‐of‐concept evidence that PC benefits a population defined explicitly by temperature‐related symptom sensitivity, distinct from the largely inferential rationale presented for the other clinical populations discussed in this review. Given the high prevalence of heat sensitivity in MS and its substantial impact on exercise participation and daily functioning, PC represents a low‐risk, nonpharmacologic adjunct with a comparatively stronger existing evidence base than the other translational applications discussed here, though further study in larger and more diverse neurological populations is warranted.

### 8.2. Age‐Related Thermoregulatory Decline

While PC itself has not yet been tested in older adults, this population demonstrates decreased cutaneous vasodilatory capacity, reduced sweat responses, and decreased thermoreceptor sensitivity compared with younger individuals [60–62], physiological changes that provide a mechanistic basis for exploring PC’s potential benefit in this group. Several studies report a delayed core temperature threshold for sweating in older adults, along with reduced evaporative heat loss secondary to lower overall sweat rates. The age‐related reduction in sweating is driven not only by delayed onset but also by decreased sweat production per gland, potentially reflecting sweat gland atrophy or reduced cholinergic sensitivity [63–65]. These impairments are particularly evident during exercise in the heat, where older adults exhibit diminished local sweat rates at comparable exercise intensities relative to younger individuals [66].

In addition, aging is associated with impaired heat‐induced increases in SkBF [27, 56]. Older adults display blunted increases in SkBF for an equivalent rise in core temperature and reduced time‐dependent vasodilatory responsiveness, when matched for fitness, hydration status, and acclimation to their younger counterparts [6, 67]. During exercise in the heat, rising metabolic heat production necessitates concurrent increases in SkBF and active skeletal muscle blood flow. This dual demand challenges CV stability, particularly in older individuals with reduced vascular compliance and cardiac reserve. Maintenance of arterial pressure requires redistribution of blood flow away from renal and splanchnic beds toward active muscle and skin, alongside increases in CO [6, 49, 68]. However, cutaneous vasodilation is normally constrained to prevent excessive reductions in central blood volume and arterial pressure. The more pronounced attenuation of SkBF during exercise in older adults may therefore represent a compensatory limitation aimed at preserving blood pressure, although at the expense of effective heat dissipation [69].

Many studies have suggested that exercise in the elderly population may lead to gradual improvement in exercise capacity and heat intolerance [65, 70, 71]. Given the age‐related thermoregulatory changes discussed above, interventions that modestly reduce thermal strain without imposing additional circulatory demands may therefore be of further benefit in this population. PC targets highly vascularized glabrous skin surfaces that facilitate heat exchange and may assist in peripheral heat removal without requiring large increases in systemic vasodilation. In older adults with limited ability to augment CO or redistribute blood flow, even small reductions in core or skin temperature could theoretically lessen CV strain and improve with‐session exercise tolerance.

### 8.3. Cardiopulmonary Disease Populations

Although no trials have yet evaluated PC in cardiopulmonary disease populations, patients with heart failure, pulmonary hypertension, chronic obstructive pulmonary disease, and those recovering from myocardial infarction frequently demonstrate impaired thermoregulatory capacity during exertion, raising the theoretical possibility that PC could offer a similar benefit in this group. The primary abnormality in these patients is a blunted SkBF response with a preserved or comparable sweating response relative to healthy controls [67, 72–75]. For example, in patients with congestive heart failure, several investigations have reported significantly reduced cutaneous vascular conductance during both passive and exercise‐induced heat stress, suggesting a diminished capacity for heat dissipation, especially during bouts of exercise [72–74]. These alterations may reflect dysfunctional sympathetic regulation, vascular remodeling, endothelial impairment, and reduced peripheral vasodilatory reserve [50, 72, 74].

Importantly, during both passive and exercise‐induced thermal stress, individuals with heart failure exhibit greater whole‐body heat storage than age‐matched controls despite similar metabolic heat production [73, 74]. This disproportionate heat retention likely reflects impaired transfer of heat from the core to the periphery, resulting in greater thermal strain for a comparable workload. Elevated internal temperature may further amplify sympathetic activation, increase heart rate, and accelerate CV drift, thereby compounding hemodynamic instability and reducing overall exercise tolerance [7, 8, 42]. Importantly, in states of limited cardiac reserve, even modest increases in cutaneous vascular conductance can meaningfully reduce ventricular preload and arterial pressure, thereby amplifying perceived exertional intolerance [6, 48]. Such hemodynamic constraints are not unique to heart failure and likely extend to cardiopulmonary populations with reduced cardiac reserve, pulmonary vascular limitation, or impaired autonomic function. In these settings, thermal strain may further compromise stroke volume and arterial pressure control, thereby lowering the threshold for exertional intolerance in this population.

PC represents a physiologically targeted adjunct that may mitigate these limitations. By enhancing peripheral heat extraction without requiring further systemic vasodilation, PC may reduce whole‐body heat storage, attenuate reflex sympathetic activation, and preserve central hemodynamic stability during exertion. In doing so, it may improve tolerability of physical activity, extend submaximal work duration, and potentially enhance participation in structured or home‐based exercise interventions, a recognized limitation of current cardiac rehabilitation programs [76]. Integration of PC into the existing rehabilitation programs may be feasible, as it could be applied during supervised sessions without substantially altering established exercise protocols (Figure 3). Importantly, the proposed benefit of PC is not direct disease modification but enhancement of training stimulus. Exercise‐based rehabilitation reduces mortality and improves functional capacity in patients with cardiopulmonary disease [77], and gains in cardiorespiratory fitness are themselves independently associated with reduced CV and all‐cause mortality [78, 79]. By improving within‐session tolerability and supporting greater cumulative training participation, PC may carry meaningful downstream prognostic implications, even in the absence of a direct cardioprotective effect.

**FIGURE 3:**
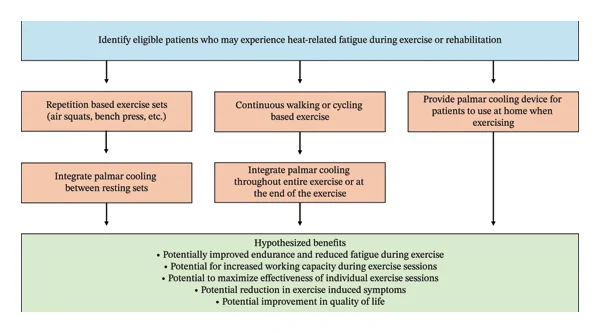
Proposed framework for incorporating palmar cooling into existing rehabilitation exercise plans. Anticipated benefits are hypothesized based on mechanistic rationale and require clinical validation in these populations.

### 8.4. Postsurgical Rehabilitation

The postoperative recovery period introduces multiple physiological stressors that may impair a patient’s ability to regulate body temperature effectively [80], and although this represents a theoretical extension rather than a directly studied application of PC, the underlying mechanisms merit consideration. Surgical trauma initiates an acute systemic inflammatory response characterized by increased circulating cytokines, including interleukin‐1, interleukin‐6, and tumor necrosis factor‐α. These mediators act centrally on the anterior hypothalamus to elevate the thermoregulatory set point, contributing to postoperative fever and a higher baseline core temperature [81–83]. An elevated resting temperature reduces the physiological margin before critical thermal thresholds are reached during exertion. This altered baseline may be compounded by opioid analgesics, which are commonly administered for postoperative pain control. Opioids influence central thermoregulatory pathways and can modify peripheral vascular tone, potentially altering heat dissipation responses [84, 85]. The combined effects of inflammation and pharmacologic modulation may therefore narrow thermoregulatory reserve during early mobilization and rehabilitation. Beyond managing postoperative hyperthermia, utilization of the AVAs has advantages beyond cooling and can be applied to perioperative thermal dysregulation in the Post‐Anesthesia Care Unit (PACU) increasing patient comfort and stability. Historically, the clinical utilization of glabrous skin vascular networks emerged from the inverse application of this mechanism, postanesthetic rewarming. Early foundational work demonstrated that applying negative pressure to a heated hand mechanically distended blood vessels in the hand allowing accelerated recovery from mild hypothermia via an expedited heat transfer [86]. This process utilizes the same glabrous skin heat exchange but in an inverse fashion, for thermal energy delivery rather than extraction. This serves as a highly effective, noninvasive countermeasure to postanesthetic shivering and hypothermia.

In orthopedic and other surgical populations requiring immobilization, even short periods of disuse are associated with rapid skeletal muscle atrophy and CV deconditioning [87, 88]. Beyond reductions in strength and functional capacity, physical deconditioning is associated with impaired vascular responsiveness and attenuated cutaneous vasodilatory capacity during heat stress [89, 90]. Although alterations in sweating may occur, impaired skin blood flow appears to play a more prominent role in reduced heat dissipation under deconditioned states [90]. Postsurgical patients often exhibit reduced aerobic capacity after periods of inactivity, routine rehabilitation tasks may represent a higher relative metabolic intensity compared with their preoperative baseline [91]. Higher relative intensity is associated with greater sympathetic activation and accelerated heat accumulation during exercise. When superimposed upon an elevated baseline core temperature and impaired vascular heat dissipation, this may shorten exercise duration and limit the quality of rehabilitation sessions [1, 7, 9].

Given this convergence of inflammatory, pharmacologic, and deconditioning‐related factors, strategies that modestly reduce heat accumulation without imposing additional circulatory demands may be clinically advantageous. PC represents a noninvasive intervention that has demonstrated improvements in exercise tolerance, work volume, and perceptual strain in healthy populations under conditions of thermal stress [4, 12–34]. A practical advantage in the postsurgical setting is its localized application. Unlike whole‐body immersion or cooling garments, PC can be incorporated into physical therapy sessions without interfering with surgical dressings, orthopedic bracing, or wound care protocols. Except in procedures directly involving the palmar or plantar surfaces, targeted glabrous skin cooling allows clinicians to assist peripheral heat removal while maintaining infection control and protecting healing tissues. As such, it may represent a feasible adjunct to enhance exercise tolerance during early rehabilitation.

### 8.5. Management of Heat‐Related Illness

Heat‐related illness represents a clinical spectrum ranging from exertional heat exhaustion to life‐threatening heat stroke, defined by severe hyperthermia and central nervous system dysfunction. When thermoregulatory mechanisms fail to dissipate rising metabolic or environmental heat, progressive elevations in core temperature can lead to circulatory collapse, systemic inflammation, coagulopathy, and multiorgan dysfunction [92]. Mortality in classic and exertional heat stroke remains substantial without rapid recognition and intervention, underscoring the importance of immediate temperature reduction techniques.

Rapid cooling is the cornerstone of treatment for heat stroke, with cold‐water immersion considered the most effective modality for rapidly reducing core temperature to 38°C–39°C [93]. However, full‐body immersion may be logistically difficult in many hospital settings because of equipment, monitoring, and staffing constraints. While dedicated PC devices have not been directly studied in the heat illness setting, the broader principle of glabrous skin cooling is well established in this context: current guidance supports the use of alternative, conductive, and evaporative methods when immersion is not feasible, including targeted application of cooling to highly vascular regions [93]. The thermoregulatory importance of glabrous skin surfaces, immersion of the hands and feet in cooled water may represent a practical adjunctive strategy to facilitate heat extraction while maintaining patient access and monitoring. Further study is needed to determine whether such targeted approaches, or dedicated PC devices specifically, can approximate the cooling efficiency of whole‐body immersion.

## 9. Knowledge Gaps and Future Directions

Despite a growing body of evidence supporting the efficacy of PC in healthy and athletic populations, important gaps remain, most notably the near‐complete absence of data in older adults, individuals with cardiopulmonary disease, or those recovering from medical or surgical interventions. Future investigations should prioritize adequately powered trials conducted under clearly defined and physiologically relevant heat stress conditions. Inclusion of healthy older adults and patients with cardiopulmonary disease will be essential to determine the translational value of PC and to identify subgroups most likely to benefit. Studies may be conducted as standalone mechanistic trials or integrated into cardiac, pulmonary, or postsurgical rehabilitation settings to assess real‐world feasibility. Greater standardization of cooling parameters, including surface temperature, duration, timing relative to exercise, and device design, is warranted to enable more direct comparison across future trials. Long‐term safety and potential adaptation with repeated use should also be evaluated. Most importantly, future research in clinical populations should emphasize meaningful endpoints such as exercise tolerance, symptom burden, CV stability, and rehabilitation adherence rather than performance metrics alone.

## 10. Limitations

This review has several limitations. Most included studies enrolled small samples, typically fewer than 20 participants (Table 1), limiting the study’s statistical power. Several studies further subdivide these cohorts (e.g., by sex, training status, and heat acclimation), reducing power further within each comparison. The literature is also methodologically heterogeneous in device design, cooling temperature, and timing relative to exercise. Devices have ranged from custom negative‐pressure chambers (18°C–22°C) to unpressurized gloves, plates, and cylinders (10°C–15°C), applied continuously, intermittently between sets, or only during recovery, across settings from thermoneutral laboratories to ambient heat exceeding 40°C. This heterogeneity limits direct comparison across studies and likely contributes to the inconsistent findings in Table 1, since protocols generating minimal thermal strain would not be expected to produce effects comparable to those inducing meaningful hyperthermia. Participant and outcome‐assessor blinding was similarly inconsistent. Due to cooling being readily perceptible, most trials could not blind participants, and only Brown et al. [32] employed a validated sham device. This raises the possibility that subjective outcomes (RPE, thermal comfort), and potentially some performance outcomes, reflect participant expectation rather than physiological effect alone.

As a narrative rather than systematic review, formal quantitative bias assessment (e.g., funnel plot and Egger’s regression) was not undertaken and would in any case be unreliable given the modest number of studies per outcome domain (9–13; Table 2). Qualitatively, the picture is mixed as several foundational positive trials originated from a group with a disclosed commercial interest in PC technology (Grahn and Heller, founders of AVAcore Technologies), but this is offset by a substantial body of independently conducted trials reporting null or unfavorable findings (McMahon et al. [27, 28], Walker et al. [15], Scheadler et al. [19], Maroni et al. [25], Iwata et al. [31]), including one unpublished thesis (Kramer [29]) not subject to typical publication incentives. This pattern argues against pervasive suppression of unfavorable findings, though a residual bias toward publishing positive results in higher‐impact venues cannot be excluded. Relatedly, McMahon et al. [27] and McMahon and Kennedy [28] report outcomes from the same 11 subjects and underlying dataset; both are retained here for their distinct outcome measures, but this overlap should be considered when interpreting the apparent volume of independent replication in the resistance‐training literature.

Finally, the population studied is relatively homogeneous. Most trials enrolled young, physically active, or athletically trained participants, with clinical or heat‐sensitive populations represented only by the preliminary MS work discussed above. This limits generalizability to older adults, individuals with cardiopulmonary disease, or postsurgical patients, populations for whom the mechanistic rationale for PC may be strongest but direct evidence is currently absent. Given these constraints, the certainty ratings in Table 2 should be considered provisional, and the clinical discussion throughout this review is intended to motivate future investigation rather than support immediate changes to practice in the absence of direct clinical trial evidence in the populations discussed.

## 11. Conclusions

Thermal strain is a primary determinant of exercise tolerance, CV stability, and perceptual effort during exertion. During strenuous exertion, especially in warm environments, the requirement to dissipate metabolic heat imposes substantial circulatory stress, contributing to CV drift, increased sympathetic activation, and earlier voluntary termination of exercise. PC leverages the unique vascular architecture of glabrous skin to enhance peripheral heat extraction and may attenuate these responses by slowing the rise in core temperature.

Evidence in healthy populations suggests that PC can improve endurance capacity, resistance training volume, and subjective comfort when applied under conditions of sufficient thermal load. Mechanistically, benefits appear most pronounced when heat accumulation meaningfully contributes to fatigue. Although data in clinical populations are limited, the physiologic rationale for application in older adults and cardiopulmonary disease is compelling, given their impaired thermoeffector responses and reduced CV reserve. It should be noted that direct evidence for PC in clinical populations is currently absent; the translational rationale presented here is mechanistically grounded but requires prospective clinical investigation before practice recommendations can be made. PC may represent a low‐burden adjunct to exercise and rehabilitation programs and a practical adjunctive strategy in heat illness management. However, current evidence is constrained by small sample sizes, methodological heterogeneity, and limited clinical endpoints. Well‐controlled trials in aging and disease populations, conducted under standardized thermal conditions and emphasizing clinically meaningful outcomes, are required to define the true translational value of PC.

## Author Contributions

Robert J. Heins and William E. Reisinger IV: conceptualization, investigation, and writing–original draft. Brett Norris: investigation and writing–review and editing. Robert Rogers: conceptualization, supervision, and writing–review and editing.

## Funding

The authors received no funding for the creation of this manuscript.

## Disclosure

All authors have read and approved the final version of the manuscript.

## Ethics Statement

This study is a review of previously published literature and did not require ethical approval.

## Consent

Informed consent was not applicable to this study.

## Conflicts of Interest

The authors declare no conflicts of interest.

## Supporting Information

Additional supporting information can be found online in the Supporting Information section.

## Supporting information


**Supporting Information** Supporting Information includes the complete search strategies for PubMed/Medline, Embase, and Scopus. It also contains risk of bias assessments evaluated using the RoB 2 tool for cross‐over and parallel‐group palmar cooling studies, alongside assessments for nonrandomized studies evaluated using the ROBINS‐I tool.

## Data Availability

Data sharing is not applicable to this article as no datasets were generated or analyzed during the current study.
