# Supplementary material for: Palmar Cooling as a Thermoregulatory Intervention: Mechanistic Basis and Implications for Exercise and Clinical Applications
Source: Transl Sports Med. 2026 Oct 18;2026:4204958. doi: 10.1155/tsm2/4204958 (PMC13678282; doi:10.1155/tsm2/4204958)
Supplement: Supplementary file 1 [file TSM2-2026-4204958-s001.docx]

**Appendix A: Full Search Strategies**

Last search date: August 3rd, 2026

PubMed/Medline:

("palm cooling"[tiab] OR "palmar cooling"[tiab] OR "hand cooling"[tiab] OR "glabrous skin cooling"[tiab]) OR ("cooling glove"[tiab] AND ("exercise"[tiab] OR "heat"[tiab] OR "thermal"[tiab] OR "thermoregulation"[tiab] OR "recovery"[tiab])) OR (("arteriovenous anastomoses"[tiab] OR "arteriovenous anastomosis"[tiab]) AND ("skin"[tiab] OR "glabrous"[tiab] OR "thermoregulation"[tiab] OR "temperature"[tiab] OR "heat"[tiab])) OR (("heat extraction"[tiab] OR "heat removal"[tiab] OR "pre-cooling"[tiab] OR "precooling"[tiab]) AND ("hand"[tiab] OR "hands"[tiab] OR "palm"[tiab] OR "palms"[tiab] OR "glabrous"[tiab])) AND "humans"[MeSH Terms] AND "english"[Language]

Embase:

('palm cooling':ti,ab OR 'palmar cooling':ti,ab OR 'hand cooling':ti,ab OR 'glabrous skin cooling':ti,ab) OR ('cooling glove':ti,ab AND ('exercise':ti,ab OR 'heat':ti,ab OR 'thermal':ti,ab OR 'thermoregulation':ti,ab OR 'recovery':ti,ab)) OR (('arteriovenous anastomosis'/exp OR 'arteriovenous anastomoses':ti,ab) AND ('skin':ti,ab OR 'glabrous':ti,ab OR 'thermoregulation':ti,ab OR 'temperature':ti,ab OR 'heat':ti,ab)) OR (('heat extraction':ti,ab OR 'heat removal':ti,ab OR 'pre-cooling':ti,ab OR 'precooling':ti,ab) AND ('hand':ti,ab OR 'hands':ti,ab OR 'palm':ti,ab OR 'palms':ti,ab OR 'glabrous':ti,ab)) AND 'human'/de AND [english]/lim

Scopus:

( ( TITLE-ABS-KEY("palm cooling" OR "palmar cooling" OR "hand cooling" OR "glabrous skin cooling") ) OR ( TITLE-ABS-KEY("cooling glove") AND TITLE-ABS-KEY("exercise" OR "heat" OR "thermal" OR "thermoregulation" OR "recovery") ) OR ( TITLE-ABS-KEY("arteriovenous anastomoses" OR "arteriovenous anastomosis") AND TITLE-ABS-KEY("skin" OR "glabrous" OR "thermoregulation" OR "temperature" OR "heat") ) OR ( TITLE-ABS-KEY("heat extraction" OR "heat removal" OR "pre-cooling" OR "precooling") AND TITLE-ABS-KEY("hand" OR "hands" OR "palm" OR "palms" OR "glabrous") ) ) AND LANGUAGE ( english )

**Table S1**. Palmar Cooling Studies examined using RoB 2 (cross-over trials).

| **Study** | **D1: Randomization process** | **D2: Period/carryover effects** | **D3: Deviations from intended interventions** | **D4: Missing outcome data** | **D5: Outcome measurement** | **D6: Selective reporting** | **Overall** |
| --- | --- | --- | --- | --- | --- | --- | --- |
| Grahn 2005 [4] | Some concerns | Some concerns | Low risk | Some concerns | Some concerns | Low risk | Some concerns |
| Hsu 2005 [12] | Some concerns | Low risk | Low risk | Low risk | Some concerns | Low risk | Some concerns |
| Goosey-Tolfrey 2008 [13] | Some concerns | Low risk | Low risk | Some concerns | Some concerns | Low risk | Some concerns |
| Walker 2009 [15] | Some concerns | Low risk | Low risk | Low risk | Some concerns | Low risk | Some concerns |
| Zhang 2009 [16] | Some concerns | Low risk | Low risk | Low risk | Low risk | Low risk | Some concerns |
| Kwon 2010 [17] | Some concerns | Low risk | Low risk | Low risk | Some concerns | Low risk | Some concerns |
| Grahn 2009 [18] (Protocol 1: Heat stress/fatigue) | Low risk | Low risk | Low risk | Low risk | Low risk | Some concerns | Some concerns |
| Scheadler 2013 [19] | Low risk | Some concerns | Low risk | Low risk | Low risk | Low risk | Some concerns |
| Caruso 2015 [20] | Low risk | Some concerns | Low risk | Low risk | Low risk | Low risk | Some concerns |
| Kwon 2015 [21] | Some concerns | Low risk | Low risk | Low risk | Some concerns | Low risk | Some concerns |
| Adams 2016 [22] | Low risk | Low risk | Low risk | Some concerns | Low risk | Low risk | Some concerns |
| Zochowski and Docherty 2016 [23] | Low risk | Some concerns | Low risk | Low risk | Low risk | Low risk | Some concerns |
| Adams 2017 [24] | Some concerns | Low risk | Low risk | Low risk | Low risk | Low risk | Some concerns |
| Maroni 2019 [25] | Low risk | Some concerns | Low risk | Low risk | Low risk | Low risk | Some concerns |
| O'Brien 2021 [26] | Some concerns | High risk | Some concerns | High risk | Some concerns | Low risk | High risk |
| McMahon et al. 2023 [27] | Low risk | Low risk | Low risk | Low risk | Low risk | Low risk | Low risk |
| McMahon and Kennedy 2023 [28] | Low risk | Low risk | Low risk | Low risk | Low risk | Low risk | Low risk |
| Kramer 2023 [29] | Some concerns | Low risk | Low risk | Low risk | Some concerns | Some concerns | Some concerns |
| Iwata 2024 [31] | Low risk | Some concerns | Low risk | Low risk | Low risk | Some concerns | Some concerns |
| Wydotis 2026 [33] | Low risk | Some concerns | Low risk | Low risk | Low risk | Some concerns | Some concerns |
| Wang 2026 [34] | Low risk | Low risk | Low risk | Low risk | Low risk | Some concerns | Some concerns |

**Grahn et al. (2012) reported multiple independent experimental protocols with distinct study designs; therefore, each protocol was assessed separately using the appropriate risk of bias tool.*

**Table S2**. Palmar Cooling Studies examined using RoB 2 (parallel-group).

| **Study** | **D1: Randomization process** | **D2: Deviations from intended interventions** | **D3: Missing outcome data** | **D4: Outcome measurement** | **D5: Selective reporting** | **Overall** |
| --- | --- | --- | --- | --- | --- | --- |
| Grahn 2012* [18] (Protocol 2C: Bench press training) | Some concerns | Low risk | Low risk | Some concerns | Low risk | Some concerns |
| Brown 2025 [32] | Low risk | Some concerns | Some concerns | Some concerns | Low risk | Some concerns |

**Grahn et al. (2012) [18] reported multiple independent experimental protocols with distinct study designs; therefore, each protocol was assessed separately using the appropriate risk of bias tool.*

**Table S3**. Palmar Cooling Studies examined using ROBINS-I (non-randomized).

| **Study** | **D1: Confounding** | **D2: Selection of participants** | **D3: Classification of interventions** | **D4: Deviations from intended interventions** | **D5: Missing data** | **D6: Measurement of outcomes** | **D7: Selective reporting** | **Overall** |
| --- | --- | --- | --- | --- | --- | --- | --- | --- |
| Grahn 2012* [18] (Protocol 2A: Experienced pull-up training) | Serious | Low | Low | Low | Low | Low | Low | Serious |
| Grahn 2012* [18] (Protocol 2B: Naive pull-up training) | Moderate | Low | Low | Low | Low | Low | Low | Moderate |
| Grahn 2012* [18] (Protocol 3: strength training response) | Serious | Moderate | Low | Low | Moderate | Low | Low | Serious |
| Park and Kim 2023 [30] | Serious | Low | Low | Low | Low | Moderate | Low | Serious |

**Grahn et al. (2012) [18] reported multiple independent experimental protocols with distinct study designs; therefore, each protocol was assessed separately using the appropriate risk of bias tool.*
